# Accuracy of Implant Placement Utilizing Customized Patient Instrumentation in Total Knee Arthroplasty

**DOI:** 10.1155/2013/891210

**Published:** 2013-09-16

**Authors:** William D. Bugbee, Hideki Mizu-uchi, Shantanu Patil, Darryl D'Lima

**Affiliations:** ^1^Division of Orthopaedic Surgery, Scripps Clinic, 10666 North Torrey Pines Road, MS116, La Jolla, CA 92037, USA; ^2^Shiley Center for Orthopaedic Research & Education at Scripps Clinic, 11025 North Torrey Pines Road, Suite 200, La Jolla, CA 92037, USA

## Abstract

Customized patient instrumentation (CPI) combines preoperative planning with customized cutting jigs to position and align implants during total knee arthroplasty (TKA). We compared postoperative implant alignment of patients undergoing surgery with CPI to traditional TKA instrumentation for accuracy of implant placement. Twenty-five consecutive TKAs using CPI were analyzed. Preoperative CT scans of the lower extremities were segmented using a computer program. Limb alignment and mechanical axis were computed. Virtual implantation of computer-aided design models was done. Postoperative coronal and sagittal view radiographs were obtained. Using 3D image-matching software, relative positions of femoral and tibial implants were determined. Twenty-five TKAs implanted using traditional instrumentation were also analyzed. For CPI, difference in alignment from the preoperative plan was calculated. In the CPI group, the mean absolute difference between the planned and actual femoral placements was 0.67° in the coronal plane and 1.2° in the sagittal plane. For tibial alignment, the mean absolute difference was 0.9° in the coronal plane and 1.3° in the sagittal plane. For traditional instrumentation, difference from ideal placement for the femur was 1.5° in the coronal plane and 2.3° in the sagittal plane. For the tibia, the difference was 1.8° in the coronal plane. CPI achieved accurate implant positioning and was superior to traditional TKA instrumentation.

## 1. Introduction

Accurate alignment and positioning of implants in total knee arthroplasty (TKA) is an important goal of the procedure. Numerous studies have demonstrated a high frequency of implant malalignment in TKA, regardless of the surgical techniques utilized [1–7]. The innovation cycle of TKA has mirrored this fundamental concept. Initially, free-hand surgical cuts were performed prior to the placement of implant components. Subsequently, mechanical alignment guides were devised based on bony or external landmarks, and predetermined angular or measured resections were performed. More recently, image-guided or imageless computer navigation systems have been developed to guide the surgical procedure and ultimate component alignment. The most recent innovation in TKA is customized patient instrumentation (CPI), which has been introduced as a next generation technology in an effort to further improve the accuracy and precision of surgical technique, implant placement, and alignment. The concept of CPI revolves around the use of preoperatively obtained imaging studies such as plain radiographs, magnetic resonance imaging (MRI), or computed tomography (CT) scans that are then manipulated in software programs to generate three-dimensional models of an individual patient's knee anatomy and limb alignment. This model is utilized to create a customized surgical plan, which defines the surgical cuts of the tibia and femur. This surgical plan is used to create customized cutting jigs that uniquely fit the individual patient's anatomy. 

The purpose of this study was to determine if a CT-based customized patient instrumentation system resulted in accurate implantation of tibial and femoral components in TKA and to compare the CPI protocol to standard mechanical alignment guides used in traditional TKA with respect to femoral and tibial component alignment. 

## 2. Materials and Methods

### 2.1. Patients

**Figure 1:**
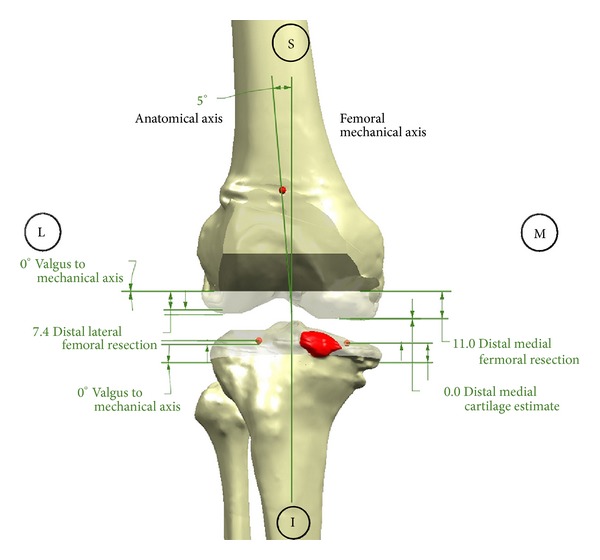
Portion of the surgical plan generated from preoperative CT scanning.

**Figure 2:**
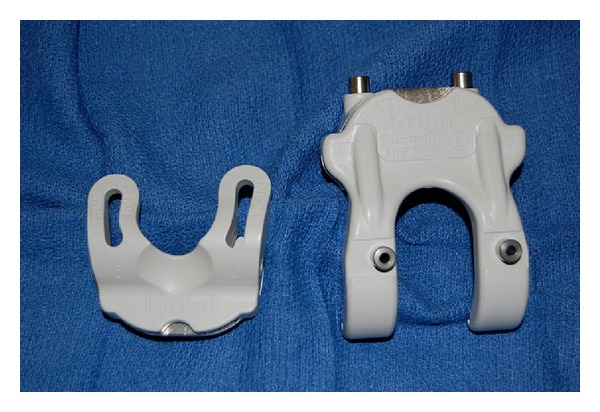
Tibial (left) and femoral (right) customized cutting guides.

**Figure 3:**
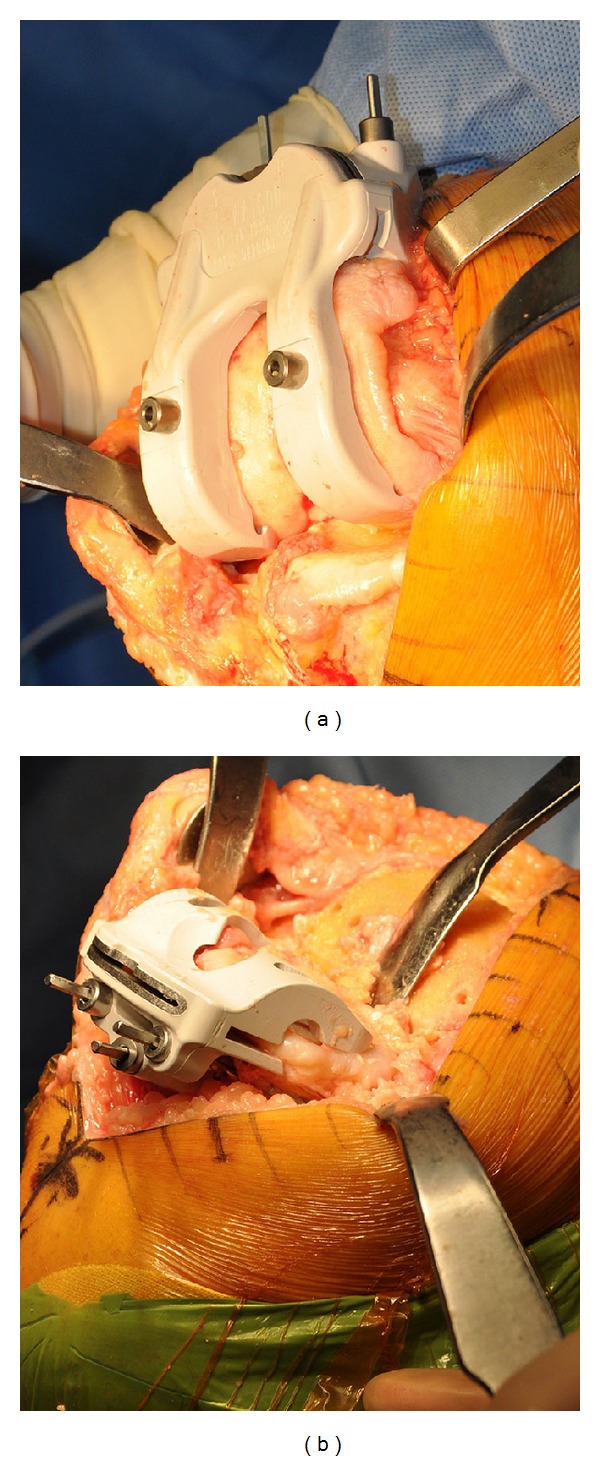
(a) Femoral cutting guide after placement on the distal femur. (b) Tibial cutting guide after placement on the proximal tibia.

With the approval of our institutional review board, we retrospectively analyzed 25 consecutive patients who underwent TKA using CT-based CPI (TruMatch, Depuy, Warsaw, Indiana). Preoperative CT scans were obtained per the device manufacturer's protocol and were used to generate a surgical plan based on predefined surgeon preferences as well as restoration of ideal mechanical alignment (Figure 1). Customized cutting guides were then made and delivered sterile to the operating room (Figure 2). Standard TKA was performed utilizing custom cutting blocks rather than traditional mechanical jigs (Figures 3(a) and 3(b)).

A second cohort of matched patients who underwent TKA using traditional instrumentation was analyzed for comparison. In the traditional TKA cohort, the targeted ideal placement was defined as 90° to the mechanical axis in the coronal and sagittal views for the femur and 90° in the coronal view for tibia. All patients underwent a standard postoperative rehabilitation protocol and were followed at 3 months, 6 months, and 1 year postoperatively. At the time of the latest clinical followup, postoperative radiographs (coronal and sagittal views) were obtained. 

### 2.2. Accuracy of Implant Placement

**Figure 4:**
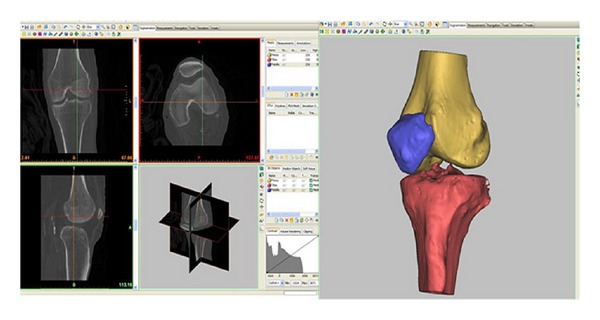
Segmenting process rendering CT scan into 3D model of the knee.

**Figure 5:**
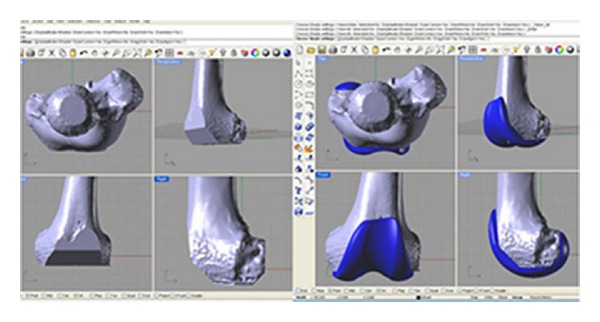
Virtual implantation of TKA based on ideal planned component position.

Preoperative CT scans of the patient's lower extremity were segmented using MIMICS 13.0 (Materialise, Belgium) (Figure 4). Three-dimensional (3D) models of the knee joint and the bones were created from which limb alignment and mechanical axis were computed for each joint. The mechanical axis of the un-operated limb was calculated by a line joining the center of the femoral head to the middle of the talar dome. The models were then imported into a modeling and design software Rhino (McNeel North America, Seattle, WA). Based on the preoperative planning protocol, virtual implantation of the TKA computer-aided design (CAD) models was performed using Rhino (Figure 5). This constituted the “ideal” planned position of the femoral and tibial components.

**Figure 6:**
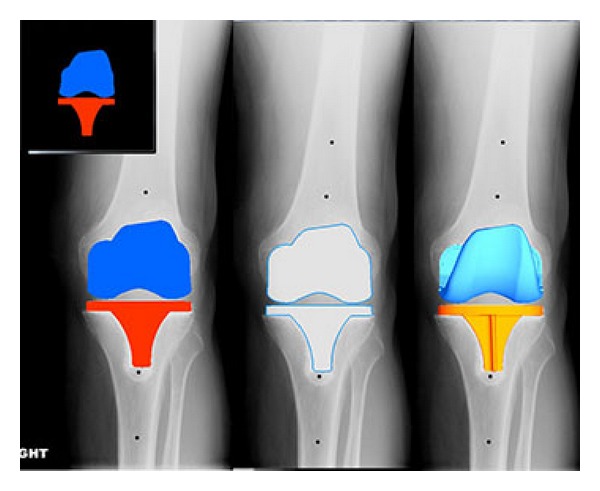
Comparison of actual versus planned surgical implantation.

The digitized postoperative knee radiographs were analyzed to determine the final placement of the actual implants. To convert the two-dimensional radiographs to 3D, an open source image matching software JointTrack (University of Florida) was used. The relative positions of the femoral and tibial implant were thus obtained from the radiographs. This data was then imported into Rhino to accurately determine the position of implanted components. This position was compared with the virtual surgical placement. The difference in planned and actual placement of the implants was calculated in the sagittal and coronal planes for the CPI group (Figure 6). In the traditional TKA group, the targeted alignment was 90° in both planes for femur and 90° in coronal plane for the tibia in sagittal view. In the traditional TKA group, implant positions were calculated in the same manner as for the CPI group. 

### 2.3. Statistics

Statistical analysis included *t*-test for equality of means and was determined using SPSS 13 (IBM software, Armonk, NY).

## 3. Results

In the CPI group, the mean absolute difference from the planned femoral placement was 0.8° (±0.6°) in the coronal plane and was 1.2° (±0.9°) in the sagittal plane (Table 1). The single outlier in this group (–4.4° difference) was a patient with posttraumatic femoral deformity, which necessitated a deviation from the planned protocol intraoperatively. In the traditional TKA group, this difference from ideal placement was 1.5° (±1.6°) in the coronal plane and 2.3° (±1.3°) in the sagittal plane. For the tibial tray, the difference from planned placement was 1.0° (±1.0°, range, –1.6° to 1.7°) in the coronal plane for the CPI group, while in the traditional TKA group, the difference from ideal placement was 1.8° (±1.6°, range, –3° to 6.4°) in the coronal plane. The comparison between the CPI and the traditional TKA group was statistically significant for femoral implant positioning (*P* < 0.01) but not for the tibial implant positioning. More outliers (±3°) occurred in the traditional TKA group.

## 4. Discussion 

Interest in custom or patient-specific cutting guides is increasing. The potential for improved surgical efficiency with decreased operative times using fewer instruments and the possibility of improvements in surgical accuracy compared to conventional mechanical instruments are attractive features. Although computer-assisted techniques have demonstrated improved component alignment in TKA, the relative cost and increased operative time has led to resistance in the widespread adoption of this technique during TKA surgery [7, 8].

Few published clinical studies are available analyzing CT-based CPI for TKA. The results of our study validates the concept that using a CT-based protocol to create a three-dimensional model and subsequent cutting guides resulted in accurate surgical positioning of the TKA implants. The second question we asked was whether customized patient instrumentation was more accurate than traditional mechanical instrumentation utilizing intramedullary femoral alignment guides and extramedullary tibial guides. In our study, the CPI was more accurate than mechanical instrumentation on the femoral side and equal on the tibial side. CPI was associated with fewer outliers. A number of clinical studies have evaluated TKA alignment utilizing similar MRI-based patient-specific guides. Nunley et al. [9] evaluated 150 primary TKA using either conventional instrumentation, mechanical-axis-based patient-specific instrumentation, or kinematic-based patient-specific instrumentation. They found that these MR-based systems had a similar number of mechanical outliers as mechanical instruments and, therefore, questioned their clinical utility. Ng et al. [10] reviewed 569 TKA performed with an MRI-based patient-specific instrumentation system and found slightly better mechanical alignment and fewer outliers than in a matched group of 155 conventionally instrumented TKAs.

The question of whether a CT-based or MR-based system is superior is not yet fully clarified. The relative advantage of a CT-based system is better bony landmark resolution than MR [11] and the ability to determine limb mechanical axis. Lower cost and shorter acquisition times are also potential advantages with CT. Relative disadvantages include the use of ionizing radiation.

Certain limitations of this study should be discussed. We did not attempt to measure clinical outcome. To date, no conclusive evidence has demonstrated that either computer navigation or customized patient instrumentation leads to improved clinical outcome or implant longevity. However, many authors have demonstrated a correlation between coronal alignment and TKA failure [12, 13]. The clinical value of custom patient instrumentation has yet to be conclusively determined. Another limitation of the current study is the postoperative analysis using plain radiographs rather than CT scans. In this study, the precision of the data analysis would have been improved if patients underwent both preoperative and postoperative CT scans to obtain more accurate 2D to 3D modeling of the knee and limb.

Ongoing advances in CT-based bone modeling protocols and subsequent manufacture of customized cutting blocks should lead to further improvements in surgical precision and accuracy. Additionally, the analytical methodology used in this study may be valuable in validating the accuracy of other implant alignment systems or image-based CPI protocols and devices [14, 15]. In conclusion, CPI is a novel technique that offers the potential of increased accuracy and efficiency in TKA. The CT-based CPI used in this study (Tru Match, DePuy, Warsaw, Indiana) accurately positioned implants relative to the preoperative plan and achieved overall implant alignment better than traditional mechanical instrumentation, with fewer outliers.

## Figures and Tables

**Table 1:** Mean absolute difference between the planned (CPI) and ideal (traditional TKA) placement of the femoral and tibial components. Asterisk denote statistical significance (paired *t*-test two tailed).

	CPI cohort	Traditional TKA cohort	*P* value
	Mean (SD, range)	Mean (SD, range)	
Femur			
Coronal*	0.8° (±0.6°; −1.0° to 1.5°)	1.5° (±1.6°; −3.9° to 2.9°)	*P* < 0.01
Sagittal*	1.2° (±0.9°; −4.4° to 2.4°)	2.3° (±1.3°; −2.5° to 4.7°)	*P *< 0.001
Tibia			
Coronal	1.0° (±1.0°; −1.6° to 1.7°)	1.8° (±1.6°; −3° to 6.4°)	
